# A CRISPR/Cas9 guidance RNA screen platform for HIV provirus disruption and HIV/AIDS gene therapy in astrocytes

**DOI:** 10.1038/s41598-017-06269-x

**Published:** 2017-07-20

**Authors:** Zaohua Huang, Madahavan Nair

**Affiliations:** Department of Immunology, Institute of NeuroImmune Pharmacology, Centre for Personalized Nanomedicine, Herbert Wertheim College of Medicine, Florida International University, Miami, Florida 33199 USA

## Abstract

HIV/AIDS remains a major health threat despite significant advances in the prevention and treatment of HIV infection. The major reason is the inability of existing treatments to eradicate the multiple HIV reservoirs in the human body, including astrocytes in the human brain. CRISPR/Cas9 system is an emerging gene-editing technique with the potential to eliminate or disrupt HIV provirus in HIV reservoir cells, which may lead to a complete cure of HIV/AIDS. The key components of CRISPR/Cas9 are guide RNAs (gRNAs) which determine specific sequence targeting of DNAs. This study established a novel, simple and quick screening method to identify gRNA candidates for targeting HIV provirus in astrocytes. Briefly, stable astrocytes clones with an integrated fluorescent HIV reporter and Cas9 expression gene were generated. Various gRNAs were screened for their efficiencies against HIV provirus in these cells. Moreover, these gRNAs and Cas9 protein were successfully tested on HIV latent astrocytes without Cas9 expression to mimic clinical conditions. HIV provirus gene-editing were confirmed by cell genomic DNA PCR and fluorescent marker expression analysis. In the future, the established transgenic cells can be used for other gene-editing studies and is well-suited for high-throughput screen application.

## Introduction

Human immunodeficiency virus (HIV)/acquired immunodeficiency syndrome (AIDS) is a serious pandemic disease that remains a major threat to human health. With breakthrough advances in HIV/AIDS prevention, diagnosis and treatment, the morbidity and mortality of AIDS has decreased significantly. However, AIDS remains an incurable, chronic infectious disease due to the multiple HIV latent cells in patients’ bodies. In brief, HIV infection can be divided into active and latent infection. In most human cells, HIV infection is active infection, but in very rare human cells, latent infection can occur at very early stage^1–6^. These very small numbers of latently infected cells, called HIV reservoirs are located mainly in the brain^7–9^, peripheral blood^2, 3, 5, 10^, and lymphoid tissue^1, 11, 12^. The HIV reservoir cells include resting memory T cells in lymph nodes^1, 11, 12^; astrocytes^13–16^, microglial cells^7, 16^, and macrophages^7, 8^ in the brain, and resting memory T cells and monocytes in peripheral blood^3, 5, 10, 17^. To date, the mechanism for formation of HIV reservoirs and reactivation of the latent cells remains largely unknown. There is evidence, though, that HIV provirus in reservoir cells is silenced due to multiple factors, including integration site^18–20^, chromatin status^21, 22^, accessibility of transcription factors^22–24^, and RNA interference^25–27^. Due to low penetrance efficiency at reservoir sites, antiretroviral drugs do not reach therapeutic doses there^28–30^. Moreover, even under antiretroviral therapy (ART), approximately 30 to 50% of AIDS patients eventually develop HIV-associated neurological disorders (HAND), which are cognitive, motor and/or behavioral impairments caused by HIV infection in the human brain^31–33^. HAND can further be grouped into asymptomatic neurocognitive impairment (ANI), minor neurocognitive disorder (MND) and the most severe, HIV-associated dementia (HAD). Although the mechanism of HAND remains to be elucidated, it is generally accepted that HAND is tightly correlated with HIV infection of astrocytes^13, 14, 16^, microglial cells^7, 16^ and macrophages^7, 8^ in human brains. Neurons are believed to be resistant to HIV infection. However, the neurotoxic products released from HIV-infected brain cells seriously dysregulates neuronal function and homeostasis. Astrocytes are very important supporting cells in human central nervous system and they play critical roles in physiological and pathological conditions. For example, astrocytes are structural scaffolds and also a critical component of Blood Brain Barrier. In addition, they form tripartite synapses, release and uptake neurotransmitters, and provide energy substrates to neurons. Disrupted astrocytes fail to maintain homeostasis in pathological conditions. For example, in HIV patients, the capacity of astrocytes to maintain homeostasis is disrupted by HIV and HIV proteins^16^.

The RNA-guided Clustered Regularly Interspaced Short Palindromic Repeat (CRISPR)/CRISPR-associated protein 9 (Cas9) system was derived from the adaptive immune system from bacteria. However, the current CRISPR/Cas9 system is much simpler and adapted for mammalian genome editing. The Cas9 is bioengineered for better nucleus localization and mammalian cell expression^34, 35^. The original two RNAs (crRNA and tracRNA) were converted into a single guided RNA (gRNA) in most cases^34, 35^. In detail, Cas9 has two nuclease activity domains which are named HNH and RuvC. Each of these two domains can cleave a DNA strand directed by a gRNA complementary to the target DNA sequence^34, 35^. The prerequisite to be a target sequence is the presence of a NGG sequence (protospacer adjacent motifs, PAM) at the 3′ side of the target site^35–37^; the length of the target sequence is generally 20 nucleotides long^34, 35^. In the cell nucleus, Cas9, gRNA and target DNA form a complex. HNH and RuvC domain of Cas9 each cleave a DNA strand. The double strand breaks are consequently repaired mainly by two approaches: 1. Non-homologous end-joining (NHEJ) when there is no template; 2. Homology directed repair (HDR) when there is a homogenous repair template present. NHEJ usually results in insertion or deletion, altogether called indel, while HDR results in correct repair as directed by the template^34, 35^.

The ultimate cure for HIV/AIDS will be the removal or disruption of integrated HIV provirus in latently infected cells or the elimination of these latent cells completely. However, until recently, gene therapy for HIV/AIDS has progressed very slowly. The breakthrough of gene-editing technology raises the hope for eradication of HIV because the provirus may be efficiently manipulated in the host cell genome through the newly developed technique of CRISPR/Cas9 system. As early as 2013, when the CRISPR/Cas9 technique emerged, there was a pilot application of this technique to HIV/AIDS treatments^38^. There are many progress of CRISPR/Cas9 application to the eradication of HIV provirus in HIV reservoir cells, including *in vitro*, *ex vivo* and in animal models^38–43^. However, only very limited gRNA sequences have been tested in each study. Because there are thousands of possible gRNA targeting sites available in the about 10 kb HIV provirus, therefore, the overall significance of the selected gRNAs are hard to evaluate accurately. Furthermore, in HIV gene therapy field, there are no simple and quick screening methods for the identification of strong gRNA candidates for the removal or disruption of HIV provirus from HIV reservoir cells. This paper has addressed the need for a robust gRNA screen method in HIV gene therapy field. Molecular and cell biology approaches were applied to establish an *in vitro* screen platform based on a newly created transgenic astrocyte with a HIV reporter and Cas9 expression gene. As far as we know, this paper is the first to apply a bioengineering approach to establish a brain cell line gRNA screen platform. This platform can be very helpful for researchers to screen and test the efficiencies of gRNA candidates for their gene-editing treatment of HIV latent cells.

## Materials and Methods

### Establishment of stable Cas9 expression astrocytes and two types of latent astrocyte clones

Astrocyte cell line U87 MG was purchased from ATCC, Manassas, VA. Cas9 construct of pSpCas9 (BB)-2A-Puro (PX459) were purchased from Addgene, Cambridge, MA. The construct linearization was done using BbsI (New England Biolabs, Ipswich, MA). The intact or linearized vectors were cleaned after the digestion before used for transfection. Transfection of astrocytes was done by electroporation methods using BIORAD GenePulser Xcell system (BIORAD, Hercules, CA), and transfected cell clones were selected by puromycin resistance (Life technologies, Carlsbad, CA) at 0.31 ug/ml. The clones were first expanded and verified by PCR genotyping. Primers were as follows: CasF, GCTAGTCCGTTTTTAGCGCG; CasR, AGAGTGAAGCAGAACGTGGG. The Cas9 protein expression in selected clones was confirmed by SDS-PAGE and Western blot assay. One Cas9 astrocyte clone was infected with VSVG pseudotyped RGH (Red –Green-HIV1, a doubly fluorescent HIV1 reporter). Several latent RGH integrated Cas9 clones were selected and expanded after long time culture. The integration of the HIV reporter was confirmed by genomic DNA PCR and red fluorescent protein expression. These clones were named astrocyte-RGH-Cas9 (ARC). In another experiment, astrocytes were infected with VSVG pseudotyped RGH. Several latent RGH integrated clones were selected and expanded after long time culture. The integration of the HIV reporter was confirmed by genomic DNA PCR and red fluorescent protein expression. These clones were named RGH-astrocyte (RGH-As) and they did not express Cas9 protein. The PCR primers to check the integration of fluorescent HIV reporter in above two types of clones were as follows: 1. mCherry region: CheF1, CACTACGACGCTGAGGTCAA; CheR1, CCAGGTCTCGAGCCTACTTG; CheF2, CCTGTCCCCTCAGTTCATGT; CheR2, CCCATGGTCTTCTTCTGCAT. 2. LTR region: LTRF1, ATCCACTGACCTTTGGATGG; LTRR1, GTACTCCGGATGCAGCTCTC; LTRF2, GGCTAATTCACTCCCAACGA; LTRR4, CTCAGGGTCATCCATTCCAT.

### Protein samples analysis by SDS-PAGE and Western Blot

The total protein of astrocytes was extracted with RIPA buffer and protease inhibitors cocktail from Fisher Scientific, Pittsburgh, PA. After 4–15% gradient SDS-PAGE, the proteins were electro-transferred to Immobilon-P, PVDF membrane (Millipore Corp., Bedford, MA). Nonspecific protein binding sites on the membrane were blocked with nonfat dry milk. Primary monoclonal antibody of Cas9 from Fisher scientific and primary antibody of GAPDH from Sigma (St. Louis, MO) were used. After washing, the blots were incubated with the appropriate horseradish peroxidase-conjugated secondary antibody (Promega, Madison, WI). Blots were washed again and Supersignal West Pico chemiluminescence kit (Thermo Scientific, Waltham, MA) was used to detect target proteins. A BioRad ChemiDoc MP imaging system with Image Lab software was used to catch the images.

### Cas9 protein intracellular localization by Immunocytofluorescence experiment

Cells were cultured in the chamber slides (Thermo Scientific, Waltham, MA) until 60% confluency. Culture media was removed from the cell, and cold PBS was used to wash the cells twice. The cells were fixed in 4% formaldehyde in PBS at 4 °C for 15 min, after which the cells were treated with 0.5% Triton X-100 in PBS. The fixed cells were washed with PBS and blocked with normal goat serum in PBS with Triton (PBST). The primary antibody incubation was usually 1 hr, and cells were washed. The secondary antibody conjugated with Alexa488 was incubated with the cells for 1 hr and washed. The cell nuclei were counter stained with DAPI. Images were taken by a Zeiss Axio Observer fluorescence microscope (Carl Zeiss; Thornwood, NY, USA). Images were collected using ZEN2 2011 (blue edition) software (Carl Zeiss).

### VSVG pseudotyped RGH production and infection

Hek293T cells were transfected with 10 µg RGH plasmid (NIH AIDS reagent program)^44^ and 15 µg VSVG plasmid (NIH AIDS reagent program) using lipofectamine 2000 (Invitrogen, Carlsbad, CA), incubated overnight, then fresh media was used to replace old media. After 24 to 48 hours incubation, cell culture supernatant which contained VSVG pseudotyped RGH were harvested, and FBS was added to make final concentration 20%, then the virus solution was filtered through a 0.45 µm pore size Millipore filter and stored at −80 degree. Astrocytes were seeded at 1 × 10^5^ cell/well at 6-well plate and incubated overnight. Next day morning, fresh media was added to replace the old media and incubated 2 to 3 hrs. A virus solution with polybrene at final concentration of 8 µg/ml was added to the cells. After further incubation of 24 to 48 hours, cell infection status were examined under IX51fluorescence microscope of Olympus (Olympus, Tokyo, Japan).

### HIV provirus gene targeting by CRISPR/Cas9 and gRNAs in astrocytes

HIV provirus targeting sites for CRISPR/Cas9 system were designed by using a free online bioinformatics software WU-CRISPR (http://crispr.wustl.edu/cgi-bin/gRNA_predict/custom_gRNA.cgi). The off-target effects of gRNAs against human genome were investigated by another free online bioinformatics software CRISPRdirect (http://crispr.dbcls.jp). All the gRNAs chosen were predicted have no off-target in human genome by CRISPRdirect. The selected gRNA sequences were targeting LTR, nef, pol and *tat* gene regions. The sequences of the target regions are: gLTR (gL), CCGCCTAGCATTTCATCACG; g*nef* (gN), CTGGCTAGAAGCACAAGAGG; g*tat* (gT), ACCCACCTCCCAACCCCGAG; g*pol* (gP), CAGTACAATGTGCTTCCACA. These gRNAs were used either alone or in combinations of two: (gLTR and g*pol*, in brief, gL/gP; gLTR and g*tat*, in brief, gL/gT; g*nef* and g*pol*, in brief, gN/gP; g*nef* and g*tat*, in brief, gN/gT) to transfect ARC cells. After 48 hours, the transfected cells and control cells were seeded into black 96 well plates to read the levels of red fluorescent protein expression by Biotek plate reader (Biotek, Winooski, VT). The background was subtracted from each read, and the fluorescent protein level of the control group was normalized as 100%. The deletion of HIV provirus with combinations of gN and gP; gN and gT was examined by gN and gP deletion primers: DelPNF1, CTGGATGTGGGTGATGCATA; LTRR4, CTCAGGGTCATCCATTCCAT; by gN and gT deletion primers: DelTNF1, GGCAAGTTTGTGGAATTGGT; LTRR4, CTCAGGGTCATCCATTCCAT.

### Cas9 and gRNAs complex treatment of RGH-As cells for HIV provirus gene-editing

Cas9 protein was diluted into a working buffer (20 mM HEPES, 150 mM KCI, 5% Glycerol, 1 mM DTT, pH 7.5). RNA oligos (crRNA and tracrRNA) were dissolved in nuclease-Free TE Buffer, pH 7.4 and annealed in equimolar concentrations in a sterile microcentrifuge tube. Annealed RNA oligos and Cas9 proteins were incubated together to form a complex for 5 min. The complex was mixed with lipofectamine RNAiMax (Thermo Scientific, Waltham, MA) according to manufacturers’ guide. Then the complex was added to the astrocytes for transfection for 48 hrs. Cells were examined for the CRISPR/Cas9 gene-editing efficiency by fluorescence measurement and genomic DNA PCR as mentioned below. After 48 hours, the treated cells and control cells were seeded into black 96 well plates to read the levels of red fluorescent protein expression by Biotek plate reader (Biotek, Winooski, VT). The background was subtracted from each read, and the fluorescent protein level of the control group was normalized as 100%. The deletion of HIV provirus with combinations of gN and gP; gN and gT was examined by gN and gP deletion primers: DelPNF1, CTGGATGTGGGTGATGCATA; LTRR4, CTCAGGGTCATCCATTCCAT; by gN and gT deletion primers: DelTNF1, GGCAAGTTTGTGGAATTGGT; LTRR4, CTCAGGGTCATCCATTCCAT.

### Statistics processing

All experiments were repeated three times or more. Fluorescence reading of 96 well plates was represented as mean ± standard error of the mean (SEM). GraphPad Prism software was used to analyze the data using one-way analysis of variance (ANOVA) to identify the significant differences between the groups. Dunnett’s multiple comparison test was used as the post-test to compare the control group and each treatment group when there was a significant difference. Turkey multiple comparison test was used as the post-test to compare all group pairs when there was a significant difference. The results were considered significant at P ≤ 0.05.

## Results

Recently CRISPR/Cas9 system became popular in HIV/AIDs gene therapy due to its simplicity and high efficiency. The core components of this system are gRNAs and Cas9 DNA endonuclease. The essential targeting determinants of this system are the variable 20 bp short targeting nucleotides of gRNAs. There are thousands of gRNAs could be designed against 10 kb HIV provirus sequence, therefore, a screen tool is in high demand for the selection of effective and high impact gRNA for HIV provirus disruption. Currently, NeuroAIDS is a severe threat for HIV/AIDS patients and the major barrier for treating NeuroAIDs is the HIV latent cells in patients’ brains, including astrocytes, microglial cells and macrophages. This work focused on astrocytes which are the most abundant cells in the human brain.

### Establishment of stable Cas9 expression astrocyte clones

**Figure 1:**
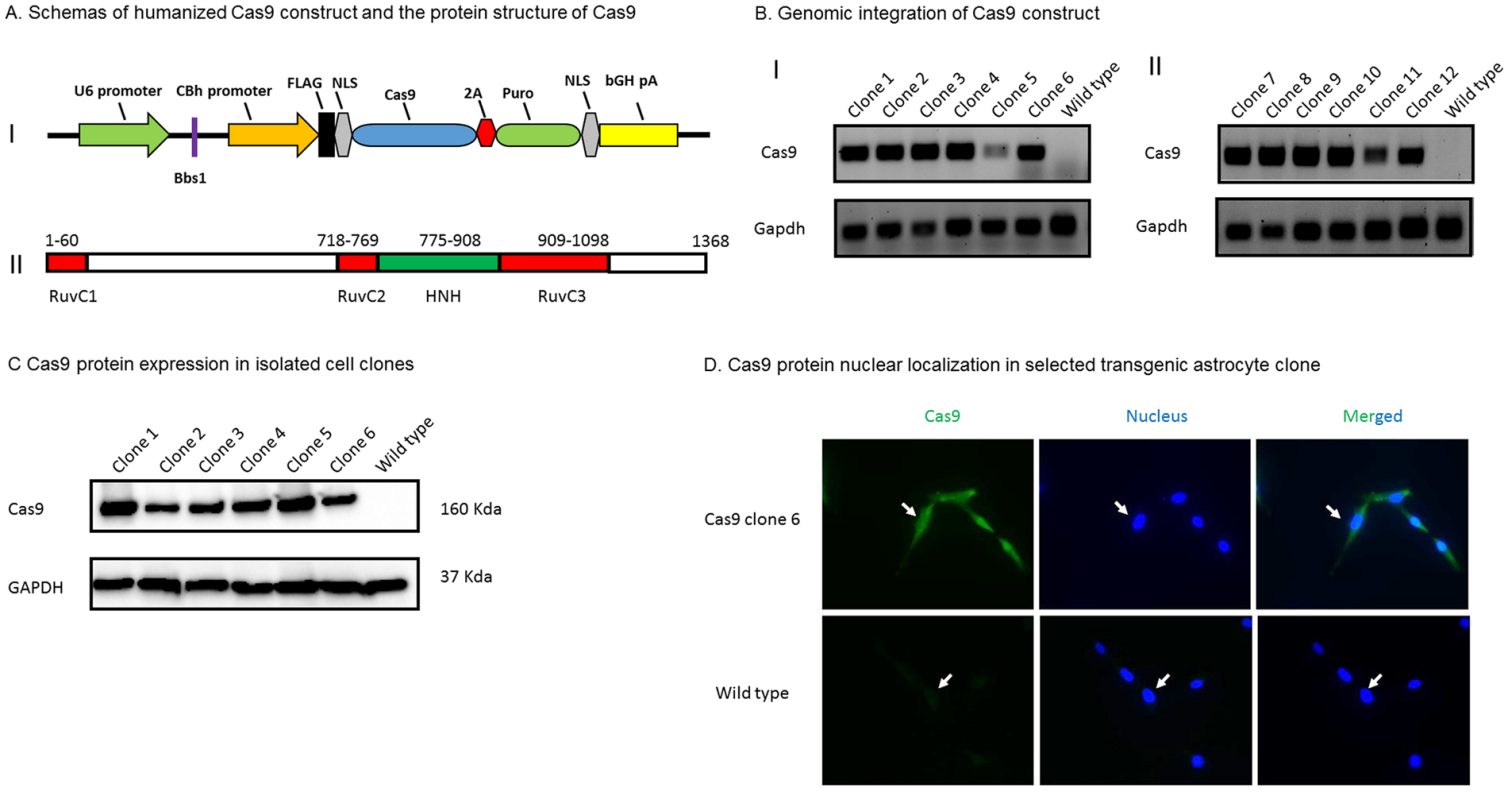
Establishment of stable Cas9 expression astrocytes. (**A**) Schemas of humanized Cas9 construct and the protein structure of Cas9. I: The structure of humanized Cas9 construct: The major DNA segments of humanized Cas9 construct were presented and their names were listed above. The digestion site of Bbs1 was marked between U6 promoter and CBh promoter. The construct encoded a humanized Cas9 protein with a flag tag and NLS domains and it conferred transfected cells the resistance to the antibiotic of puromycin. II: The domain structure of Cas9 protein: The major domains of Cas9 protein were presented and amino acid locations of each domain were listed above. (**B**) Genomic integration of Cas9 construct. Cas9 gene-specific primers were used to screen positive clones which contained Cas9 construct in cell genomes. *Gapdh* PCR products were used as a DNA extracts quality and loading control. Test results of total 12 clones were presented. (**C**) Cas9 protein expression in isolated cell clones. After the cell clone screening by PCR, protein extracts of 6 positive clones were examined with SDS-PAGE and Western blot to check protein expression of Cas9. GAPDH protein were also checked and used as a loading control. (**D**) Cas9 protein nuclear localization in selected transgenic astrocyte clone. Immunocytochemistry and fluorescence microscopy were used to determine the intracellular localization of Cas9 protein. Nucleus counterstain was used to determine the co-localization of Cas9 protein and the nucleus. The white arrows pointed to the locations of the nuclei.

To set up a strong *in vitro* screen system, we prepared an engineered stable Cas9 cell line. Since our focus was on NeuroAIDs, we chose an astrocyte cell line as our first option. We transfected astrocyte cell line cells with intact or linearized Cas9 plasmid pX 459 (Fig. 1A) and then selected Cas9 construct integrated cell clones by puromycine resistance first. After testing the death curve of astrocytes under the puromycine resistance, we chose the concentration at 0.31 ug/ml. We found that there were more positive cell clones in the linearized construct group compared to intact construct (data not shown). We expanded the cell clones and extracted cell genomic DNA for a PCR screen of Cas9 gene integration. Twelve cell clones presented a specific PCR product band while the parent cell line, named “wild type” here, did not have it (Fig. 1B). Together, we identified twelve clones which had integrated the Cas9 coding gene. After the genomic DNA PCR screen, we prepared the protein extracts of positive cell clones for Cas9 protein detection using SDS-PAGE and Western blot (Fig. 1C). The identified cell clones presented variable levels of Cas9 protein expression while wild type had no endogenous expression of Cas9 protein (Fig. 1C). To experiment further, we chose clone 6 which had moderate Cas9 protein level because we were not sure whether the highest Cas9 expression level was good or bad for the stable cells. Since Cas9 protein had to enter the nucleus to perform its gene-editing function, we checked Cas9 protein intracellular localization in our cell clone with immunocytochemistry to confirm the nucleus location of Cas9 protein. The results in Fig. 1D indicated that Cas9 protein localized in the cell nucleus according to the fluorescent nuclear counterstain.

### Establishment of HIV latent astrocyte cell model with stable Cas9 expression

**Figure 2:**
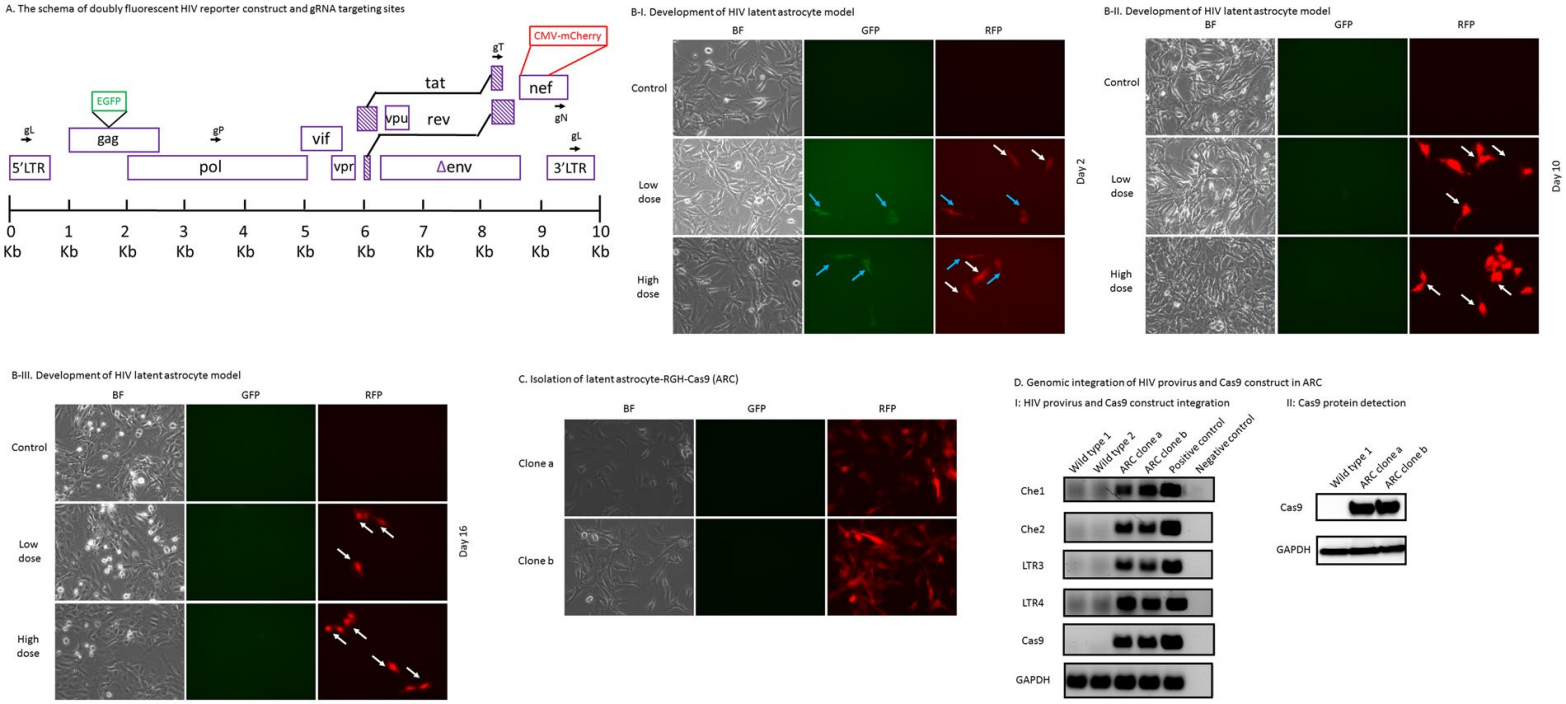
Establishment of HIV latent astrocytes with stable Cas9 expression. (**A**) The schema of doubly fluorescent HIV reporter construct and gRNA targeting sites. The DNA structure of RGH HIV reporter construct was presented and major DNA domain names were listed. A nucleotide base pair scale was provided for the segment sizes and order reference. In brief, an EGFP expression cassette was inserted into the *gag* gene and a mCherry expression cassette was inserted into the *nef* gene. In this HIV DNA genome, both *env* gene and *nef* gene were defective. This construct was not contagious and could not be replicated alone. The gRNA targeting sites were marked with black arrows and the first letter of the corresponding HIV DNA domain with a “g” prefix. gL for LTR; gP for *pol*; gT for *tat*; gN for *nef*. (**B**) Development of HIV latent astrocyte model. The isolated stable Cas9 expression clone in Fig. 1 was infected with VSVG pseudotyped RGH. A time course profile study of the fluorescent protein expression was conducted in the infected cells. In day 2, red fluorescent protein expression was either alone (shown by white arrows) or with the green fluorescent protein expression simultaneously (shown by blue arrows). In day 10 and 16, red fluorescent protein only expression (shown by white arrows) indicated the latent status of the HIV genome in these cells. (**C**) Isolation of latent astrocyte-RGH-Cas9 (ARC). Latent cell clones of ARC (2 clones were shown here) which possessed the genomic integration of both Cas9 construct and HIV provirus were isolated and expanded. Here note all the cells were expressing red fluorescent proteins. (**D**) Genomic integration of HIV provirus and Cas9 construct in ARC. I: HIV provirus and Cas9 construct integration: Multiple HIV provirus specific primers were used to verify the genomic integration of HIV provirus in selected two clones. *Gapdh* PCR products were used as a DNA extracts quality and loading control. Cas9 construct integration was also verified by Cas9 construct specific primers used in Fig. 1. II: Cas9 protein detection. After confirmation by PCR, protein extracts of ARC clones were examined with SDS-PAGE and Western blot to check Cas9 protein expression. GAPDH protein was also checked and used as a loading control.

To make a robust HIV latent cell model, we utilized a doubly fluorescent protein HIV1 reporter RGH^44^ (Fig. 2A). In brief, in VSVG pseudotyped RGH infected astrocytes, the expression of EGFP was controlled by LTR. When RGH provirus was in latent status, LTR was silent and there was no expression of EGFP, which meant no green fluorescence under a fluorescence microscopy. The expression of red fluorescent protein of mCherry was controlled by CMV promoter. Because CMV promoter was a constitutive promoter, even latently infected astrocytes were able to express red fluorescent protein all the time. Therefore, latently infected astrocytes presented only red fluorescence under a fluorescence microscopy. According to the conventional method, we infected astrocytes with VSVG pseudotyped RGH and did time-course studies of the cell infection status (Fig. 2B). In our results, after VSVG pseudotyped RGH infection, when infected cells were in active status, green fluorescent proteins of EGFP were expressed, and cells were green under fluorescence microscopy (Fig. 2B-I). When VSVG pseudotyped RGH infected cells were in latent status, only red fluorescent proteins of mCherry were expressed and cells were red under fluorescence microscopy (Fig. 2B-II;III). At early days of the infection time course, we often observed that red fluorescent protein was either expressed alone or expressed with green fluorescent protein. (Fig. 2B-I); however, after day 10, all the infected cells expressed only red fluorescent protein, which indicated the latent status of the infected cells (Fig. 2-II;III). Those infected cells expressed red fluorescent protein after many cell passages. We isolated two clones of those latent Cas9 stable astrocytes and we named these clone cells “astrocyte-RGH-Cas9 (ARC)” (Fig. 2C). Genomic DNAs of ARC were extracted for HIV provirus and Cas9 verification. We designed several PCR primer pairs specific for different regions of HIV provirus, including the mCherry coding region and LTR region. HIV provirus PCR products were detected in all of these PCRs as indicated in Fig. 2D-I. These PCR results confirmed genomic integration of RGH DNA into astrocytes’ genomes. In addition, we did Cas9 PCR to confirm that the cell line still contain integrated Cas9 expression gene (Fig. 2D-I), and we did the Gapdh PCR for the DNA quality and loading control (Fig. 2D-I). We then examined the expression of the Cas9 protein in these selected clones and we found Cas9 protein expression were still good (Fig. 2D-II).

### Screening test of gRNAs targeting HIV provirus in ARC

**Figure 3:**
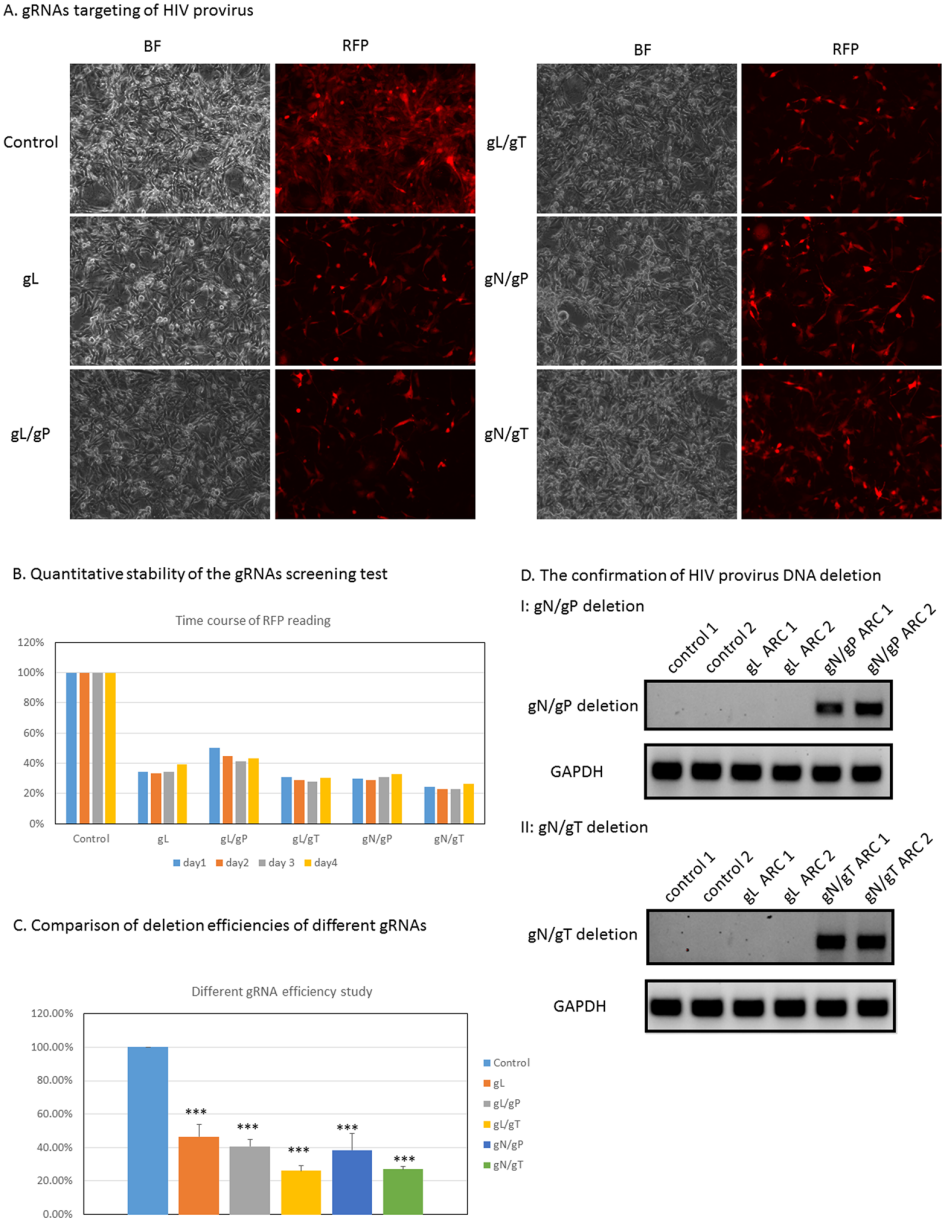
The screening tests of gRNAs in ARC. (**A**) gRNAs targeting of HIV provirus. The selected ARC clone was treated with different gRNAs which targeted different regions of HIV provirus. Successful targeting resulted in the termination of red fluorescent protein expression. Here presented a representative microscopy image of each treatment. (**B**) Quantitative stability of the gRNAs screening test. The microplate of treated ARC was read by a conventional plate reader. The red fluorescent protein expression levels were normalized by the protein level of the control group. The protein expression level of control group was presented as 100%. The 4-day readings indicated the repeatability and stability of this method. (**C**) Comparison of deletion efficiencies of different gRNAs. The microplates of different gRNA treated ARC were read by a conventional plate reader. The red fluorescent protein expression levels of different gRNA treatment groups were normalized by the protein expression level of the control group. The protein expression level of control group was presented as 100%. The figure presented the data of four different experiments. The data were expressed by mean ± SEM. Each treatment group was compared to the control by one-way ANOVA followed by Dunnett’s multiple comparison test. Note: * means P < 0.05; ** means P < 0.01; *** means P < 0.001. All two pairs comparison were conducted by one-way ANOVA followed by Turkey multiple comparison test. (**D**) The confirmation of HIV provirus DNA deletion. After gRNAs treatment, DNA samples of ARC were extracted. Specific PCR primers were used to detect HIV provirus DNA deletion in these samples. A band at a specific size indicated the positive results of DNA deletion I: gN/gP deletion presented the HIV provirus DNA segment deletion with gN and gP treatment; II: gN/gT deletion presented the HIV provirus DNA segment deletion with gN and gT treatment. Two different experimental samples were used in each test.

We had established an *in vitro* cell platform of ARC to screen gRNAs of CRISPR/Cas9 system for the removal of HIV provirus in astrocytes. The specificity and gene-editing function of CRISPR/Cas9 system depends primarily on gRNAs. As an initial test, we applied the gRNA design bioinformatics tools of WU-CRISPR (http://crispr.wustl.edu/cgi-bin/gRNA_predict/custom_gRNA.cgi) to design several gRNAs targeting LTR region, pol gene region, tat gene region and nef gene region as indicated in Fig. 2A. The off-target effects of gRNAs against human genome were investigated by another free online bioinformatics software CRISPRdirect (http://crispr.dbcls.jp). All the gRNAs chosen were predicted have no off-target against human genome by CRISPRdirect. We transfected ARC with those gRNAs either alone or in combinations of two (gL and gP; gL and gT; gN and gP; gN and gT) to delete a DNA fragment which includes the mCherry coding region. Therefore, a successful deletion should eliminate the expression of red fluorescent protein. After transfection, we took images of those cells with light and fluorescence microscopy as indicated in Fig. 3A. In Fig. 3A, compared to the control group, there were some minor morphologic changes for those astrocytes of gL/gT; gN/gP and gN/gT. The changes seemed unlikely to be related to gRNA treatment, since the other groups did not present such changes. Slight morphologic changes occurred here perhaps were caused by the low level toxicity of the transfection reagent or cell high density, but the changes seemed unlikely affect the elimination of HIV provirus segment later as shown in Fig. 3D. We observed reduced expression of red fluorescent protein in all gRNAs treated cells compared to that in the control cells (Fig. 3A). To quantify the fluorescent protein level decrease, we seeded the transfected cells to 96 well plates and used a Biotek synergy HT plate reader to obtain a quantitative measurement of the cell red fluorescent protein (Fig. 3B). The red fluorescent protein readings were very stable in a time course study when all samples were normalized to the control which was 100% (Fig. 3B). We observed significantly reduced expression of red fluorescent protein in those gRNA treated cells compared to that of the control cells (Fig. 3C), which confirmed the fluorescence microscopy observation noted previously (Fig. 3A). The order of the efficiencies from high to low was gL/gT, gN/gT, gN/gP, gL alone and it seemed that the efficiencies of gRNAs combination were better than that of gL alone even though it was not statistically different (Fig. 3C). We extracted genomic DNA samples from the control cells and gRNA treated cells and examined the deletion of HIV provirus from host genome using conventional PCR. The primer pairs was designed in such a way that only deleted genomic DNA could produce a band at a specific molecular size. In our PCR experiment design, only two combinations produced feasible PCR products: gN/gP; gN/gT. The upper panel of Fig. 3D detected the deletion from combination of gRNA targeting pol and nef only (gN/gP) as predicted, while the lower panel of Fig. 3D detected the deletion from combination of gRNA targeting tat and nef only (gN/gT) as predicted. Each test in Fig. 3D included samples from two different experiments. In brief, data from Fig. 3D clearly confirmed the successful deletion of HIV provirus fragment using two gRNAs as predicted. Furthermore, the deletion is specific since the LTR gRNA and the control group did not produce such PCR products (Fig. 3D).

### Cas9 and gRNA complex treatment of RGH-As for HIV provirus gene-editing

**Figure 4:**
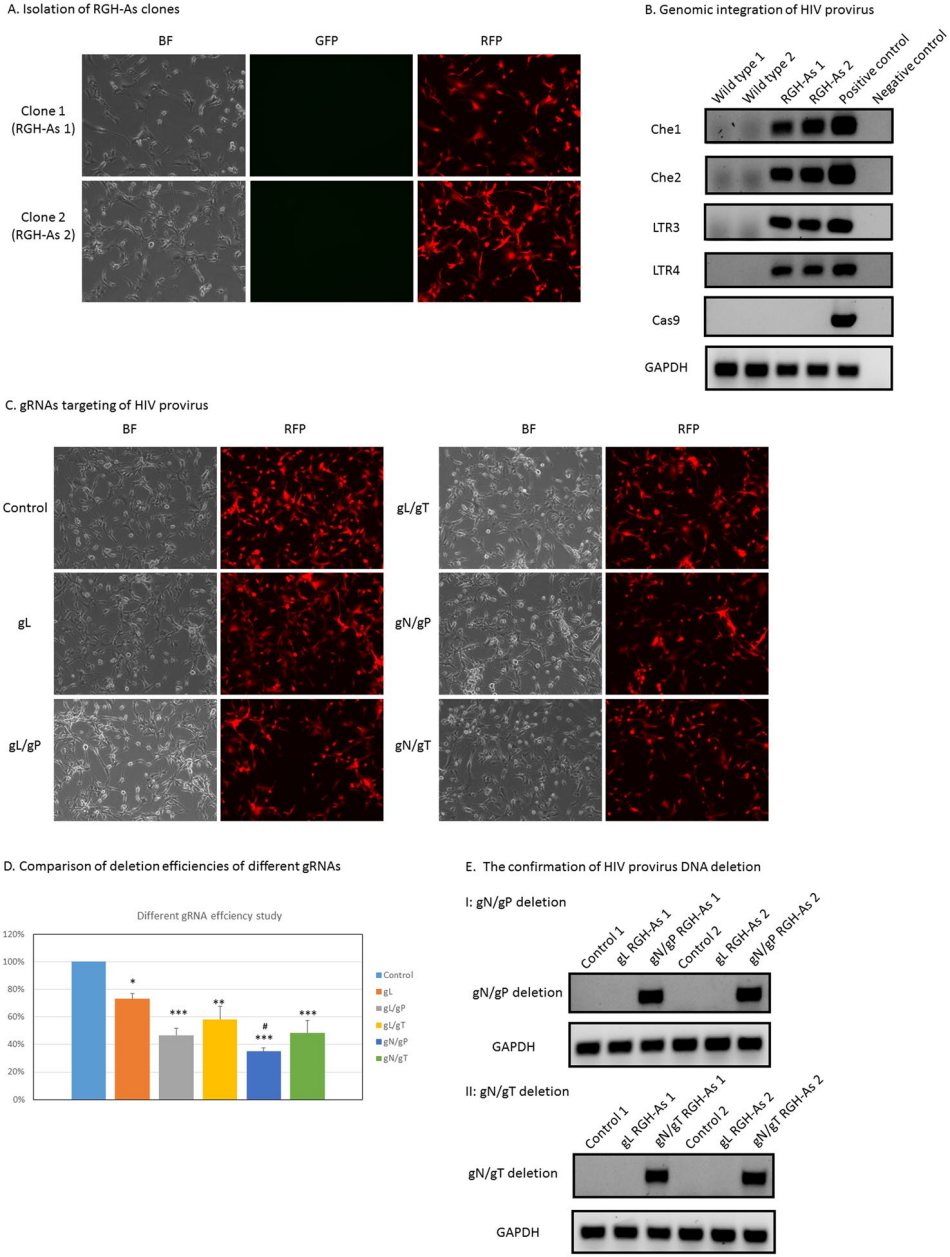
HIV provirus targeting in RGH-As by Cas9 protein and gRNAs complex. (**A**) Isolation of RGH-As clones. Wild type astrocytes were infected with VSVG pseudotyped RGH. Latent HIV astrocyte clones were isolated and examined by fluorescent microscopy. (**B**) Genomic integration of HIV provirus. Multiple HIV provirus specific primers were used to verify the genomic integration of HIV provirus in selected two clones. *Gapdh* PCR products were used as a DNA extracts quality and loading control. Cas9 construct PCR was used to confirm the absence of Cas9 construct. Two clones were used in each test. (**C**) gRNAs targeting of HIV provirus. The selected RGH-As was treated with different gRNAs which targeted different regions of HIV provirus. Successful targeting resulted in the termination of red fluorescent protein expression. Here presented a representative microscopy image of each treatment. (**D**) Comparison of deletion efficiencies of different gRNAs. The microplates of different gRNA treated cells were read by a conventional plate reader. The red fluorescent protein expression levels were normalized by the protein expression level of the control group. The protein expression level of control group was presented as 100%. The figure presented the data of four different experiments. The results were expressed by mean ± SEM. Each treatment was compared to the control by one-way ANOVA followed by Dunnett’s multiple comparison test. Note: * means P < 0.05; ** means P < 0.01; *** means P < 0.001. All two pairs comparison were conducted by one-way ANOVA followed by Turkey multiple comparison test. Note: ^#^means P < 0.05 when compared to gL group. (**E**) The confirmation of HIV provirus DNA deletion. After gRNA treatment, RGH-As DNA samples were extracted. Specific PCR primers were used to detect HIV provirus DNA deletion. A band at a specific size indicated the positive results of DNA deletion I: gN/gP deletion presented the HIV provirus DNA segment deletion with gN and gP treatment; II: gN/gT deletion presented the HIV provirus DNA segment deletion with gN and gT treatment. Two different experimental samples were used in each test.

In human gene therapy, there is no endogenous Cas9 expression in patients’ cells. In order to mimic actual clinical situations, we first established astrocyte clones with integrated HIV reporter and named those astrocytes “RGH-As” (Fig. 4A). We extracted cell genomic DNAs and verified the integration of RGH HIV reporter using multiple primer pairs which detected the coding sequence of mCherry and LTR (Fig. 4B). The specific PCR product bands clearly confirmed the integration of RGH HIV reporter. We also confirmed the RGH HIV reporter integration using fluorescent microscopy to detect red fluorescent protein expression (Fig. 4A). Then we delivered Cas9 protein and gRNA complex to those RGH-As and gRNAs are applied either alone or in combinations of two (gL/gP; gL/gT; gN/gP; gN/gT) to delete a DNA fragment which included the mCherry protein coding region. Therefore, a successful deletion should eliminate the expression of red fluorescent protein and result in no red fluorescence under fluorescence microscopy. We took images of those different groups of astrocytes with a light and a fluorescence microscopy as indicated in Fig. 4C. In the control group, all of the cells showed red fluorescence under the fluorescence microscopy (Fig. 4C). Compared to the control cells, different reduced expression of red fluorescent protein were found in gRNAs treated groups (Fig. 4C). To measure the decrease of red fluorescence, we applied BioTek plate reader to quantify the expression level of red fluorescent protein. Quantification of the fluorescent protein level confirmed a significant decrease present in all gRNA treated groups compared to that of the control group as shown in Fig. 4D. Furthermore, among all two pairs comparison, the decrease of red fluorescent protein expression in gN/gP group was significant more compared to gL group, while all other comparisons were not statistically different. To confirm the deletion of HIV provirus, we extracted cell genomic DNA samples from the control cells and gRNA treated cells and examined with specific primers to detect the deletion from gN/gP; gN/gT. As in ARC, data from Fig. 4E clearly confirmed the successful deletion of HIV provirus fragment using gRNA combinations of gN/gP and gN/gT. We detected the successful deletion of HIV provirus in those gRNAs treated RGH-As (Fig. 4E). Each PCR test in Fig. 4E included samples from two different experiments. Furthermore, the deletion is specific since samples from the gL group and the control group did not produce such PCR products (Fig. 4E). Moreover, all of the data confirmed the successful treatment of Cas9 proteins and gRNAs in RGH-As of our experiments.

## Discussion

In the United States, AIDS was first diagnosed in 1981. More than three decades later, HIV/AIDs remains a major health threat for Americans. The main barriers to a complete cure are HIV latent cells that exist in patients’ bodies. These HIV latent cells are also called HIV reservoirs. The very few latently infected cells can escape the effect of antiretroviral drugs and the attack of human immune cells. It is generally accepted that HIV latent cells caused HIV virus rebound in AIDS patients when they stop antiretroviral medications. HIV gene therapy progressed very slowly until recent breakthroughs in gene-editing technologies. CRISPR/Cas9 is one of the robust gene-editing techniques that is popular today^34, 35^ and has been utilized in HIV latent cell eradication studies^38–42^. The CRISPR/Cas9 system was derived from the adaptive immune systems of bacteria. The core components of CRISPR/Cas9 system are Cas9 nuclease and gRNAs which determine the specificity of gene-editing. There are two forms of gRNA. One form consists of two components: crRNA and tracRNA; the other form merges the two RNAs into one single gRNA, named sgRNA. In this paper, the two-component form of gRNA were used and it is novel and unique in HIV provirus gene-editing and eradication studies. The biggest advantage of the two-component gRNAs is that gRNAs can be chemically synthesized readily, applied quantitatively, and standardized easily, while the sgRNA is often applied in the form of a DNA plasmid or in the form of mRNA purified from *in vitro* transcription. It is difficult and time-consuming for sgRNA to be quantified in both ways. Furthermore, we also applied Cas9 protein instead of a Cas9 plasmid in this study. Compared to a plasmid introduction, Cas9 protein agents could be easily quantitatively applied and standardized, so our approach mimicked real clinic scenarios. As noted previously, HIV has a genome of about 10 kb while a gRNA generally only targets 20 bp of the DNA molecule, which means that there are thousands available targeting sites for HIV provirus in HIV latent cells. HIV gene therapy field is in dire need of a simple and efficient screen platform to screen gRNA candidates; our work addressed this critical gap.

Currently, most CRISPR gRNA libraries screen are used primarily for genome-wide knockout^45–49^, transcription repression^50–53^ or activation^52–54^. The gRNA libraries are usually in the form of lentiviral vectors and target all of the genes in the whole genome. Each target gene has several gRNAs. After the screen, cell clones are expanded and sequenced by next generation sequencing. The main purpose of the gRNA library screen is to find essential biological functions of specific gene or genes or to identify signal transduction pathways. There is no platform established solely for gRNA efficiency evaluation in HIV provirus eradication.

Our work established an *in vitro* astrocytes platform with a doubly fluorescent HIV reporter and stable Cas9 expression. In step one, we established a stable Cas9 astrocyte cell line; we believed this design would avoid the labor of introducing the Cas9 protein to the screen platform (Fig. 1). The stable Cas9 expression makes the cell line very fit for gRNAs-focused screening, and the synthetic two-component gRNA approach makes this method easily quantified and standardized thus facilitate a high-throughput screening establishment. According to our observation, the stable Cas9 expression was long lasting and did not affect the cell line morphology and other biological parameters (data not shown). We decided to move forward with a moderate Cas9 expressed cell line since we remain concerned about the transgenic foreign protein effect. For step two, we decided to take the advantage of a doubly fluorescent HIV reporter RGH^44^ to establish a latent astrocyte cell model in which the cell HIV infection status can be easily monitored by fluorescence microscopy observation (Fig. 2). In this case, only red fluorescent protein expression means HIV latent infection of the cells. Furthermore, this fluorescent marker can avoid the controversy of latent cell model using p24 measurement and can establish an easy way to monitor provirus status by a fluorescence microscopy and a plate reader. In addition, when we design multiplex gRNAs, we can do in such a way that a successful gRNAs combination removes a segment of HIV provirus which includes the coding sequence of red fluorescent protein. The successful application of such gRNAs will result in a subsequent loss of red fluorescence of the “cured” cell (Fig. 3). In addition, all the deletions would be able to be confirmed with both genomic DNA PCRs and fluorescence microscopy observation (Figs 3 and 4). Moreover, the efficiencies of gRNAs combination eventually could be quantified by the decrease of red fluorescent protein expression because the efficiencies of the gRNAs correlated positively to the decrease of red fluorescent protein (Figs 3 and 4). In our work, we used a conventional fluorescence plate reader to quantitatively measure the decrease of red fluorescent protein expression and it was very successful (Figs 3 and 4). Thus this platform is an accurate, simple, fast and economical approach. The time course data indicated that this method was very stable (Fig. 2). PCR product examination, reduction of red fluorescent protein reading, and fluorescence microscopy images confirmed the successful deletion of the DNA fragment of HIV provirus (Figs 3 and 4). As far as we know, this is the first gRNA screening platform established against HIV provirus using transgenic astrocytes. The biggest advantage is that the platform is also suitable for multiplex gRNA testing (Figs 3 and 4) since two or more gRNAs used concurrently guarantee the deletion of critical segments of HIV provirus in HIV latent cells. The whole setup for screening was very simple and it would not be an issue for any biomedical lab to employ this experimental system. Industry can use this method in gRNA screening and gene therapy reagent development for brain astrocyte HIV provirus removal and disruption. Furthermore, the established Cas9 cell line can be used for mechanistic studies, such as HDAC2 gene knockout employing gRNAs. The major disadvantage of this screening model is that this is just an *in vitro* cell line platform for screening. Selected gRNAs require additional testing in primary cells and *in vivo* studies. To simulate *in vivo* conditions, we tested screened gRNAs in RGH-As which do not express endogenous Cas9 (Fig. 4). We established a protocol for introducing Cas9 protein and gRNA complexes into RGH-As (Fig. 4). The results achieved are comparable to data from ARC (Figs 3 and 4). Data generated may be useful for translation into potential clinical applications in the future. Furthermore, we determined optimal quantitative parameters for introducing Cas9/gRNA complexes to disrupt integrated HIV provirus, which included Cas9 and gRNA concentrations.

Our work differs from those of other groups, such as Khalili group’s studies in several ways. For example, Khalili’s group mainly targeted the LTR region to eradicate HIV provirus in cells using the CRISPR/Cas9 system^39, 41, 55^. They used stably Cas9 and gRNAs transfected cells or applied lentivirus vector to deliver Cas9 and gRNAs, potentially eliminating major portions of HIV provirus in several latent cell models^39, 41, 55^; while here we used latently infected astrocytes model to screen various gRNAs against several regions of HIV provirus including LTR. Although the ultimate goal of CRISPR/Cas9 application in both groups is to remove or disrupt HIV provirus, our experimental focus was to develop a screening method for gRNAs, which is different from the focus of Khalili group’s study. We employed engineered ARC to screen for potential gRNA candidates for the purposes of HIV gene therapy. Our approach exploited Cas9 protein and synthetic gRNAs which were easily quantified and standardized. In fact, gRNA targeting LTR regions was used in our study as a positive control and other gRNAs combinations were used to eliminate a critical segment of HIV provirus to disrupt HIV provirus instead of its complete removal. Having multiple target sites may improve the efficiency of the CRISPR/Cas9 system, analogous to gN/gP and gL treatments comparison in Fig. 4 of this report. In our system, all gRNAs, including gRNA against LTR, achieved expected gene-editing effects on HIV provirus and caused the deletion of HIV provirus and subsequent reduced expressions of red fluorescent protein in all gRNA treated groups. However, since there were two identical LTR regions flanking the HIV provirus, one LTR gRNA action resulted in the deletion of major portion of HIV provirus and creation of one intact LTR after the DNA repair. It is impossible to design PCR primers to detect such a HIV provirus deletion unless two different gRNAs against LTR were used as in Khalili’s case^39, 41, 55^. That was why we could not present PCR data to confirm LTR targeting, but our reduced red fluorescent protein expression in gL group (Figs 3 and 4) was supporting the successful deletion of gL. Further, our results of LTR targeting were comparable to the findings of LTR targeting from Khalili’s group: we were both successful in LTR targeting using CRISPR/Cas9 system.

One limitation of this study was the missing data from off-target studies even though we performed off-target bioinformatics examination on our gRNAs using an online tool. In fact off-target effects of gRNAs were very rare as demonstrated by Khalili group’s findings^39, 41, 55^, therefore we decided to focus on efficiency rather than specificity.

In summary, by using stable transgenic cells, we have developed a novel, simple and quick screening method to identify gRNA candidates for targeting HIV provirus in astrocytes. CRISPR/Cas9 system represents a new advanced therapeutic strategy to eliminate HIV provirus from HIV latent cells, but there are many challenges in its application to treating HIV latent cells in brain. One of the biggest challenges is the delivery of Cas9 and gRNAs to the brain. Delivery of Cas9 and gRNAs to the brain will likely involve nanoparticle formulations which possess the capacity to cross the Blood-Brain-Barrier (BBB). Our research group^56–60^ has made important progress in brain drug delivery using magnetic nanoparticles (MNP) and magneto-electric nanoparticles (MENP). Studies using MNP and MENP as carriers demonstrated their feasibility to deliver loaded drugs across BBB *in vitro* and in small animals^56–60^. Their potential for targeting HIV-1 reservoir cells in brain tissue merits further study in light of their known efficiencies in brain drug delivery strategies. MNP and MENP are currently being investigated as a potential carrier for CRISPR/Cas9 system delivery to the brain in our group. Our future experiments will focus on the development of Cas9/gRNA nanoparticle formulations based on MENP and MNP and subsequent tests of those developed nanoparticle formulations *in vivo*.
